# Supplementary figures and images for: Insight into the bacterial communities of the subterranean aphid Anoecia corni
Source: PLoS One. 2021 Aug 11;16(8):e0256019. doi: 10.1371/journal.pone.0256019 (PMC8357138; doi:10.1371/journal.pone.0256019)

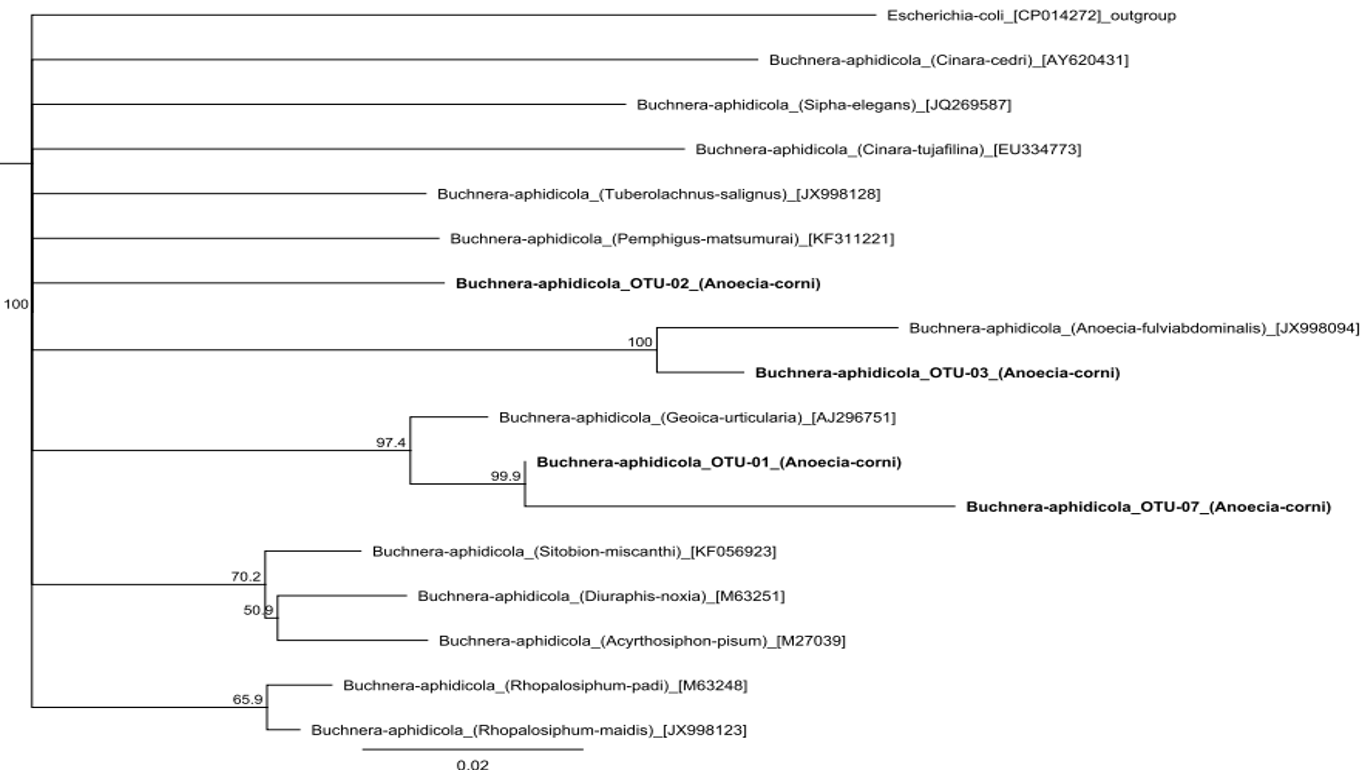

Supplement: S1 Fig — The evolutionary history was inferred using the Neighbor Joining (NJ) methods, with a J-C model. The percentage of replicate trees was verified with bootstrap of 1000 replicates. Designations in bold are strains sequenced in this study. Host names are followed by the GenBank accession number of each bacterial sequence. Geneious version 6.1 created by Biomatters. (TIF) [file pone.0256019.s001.tif]

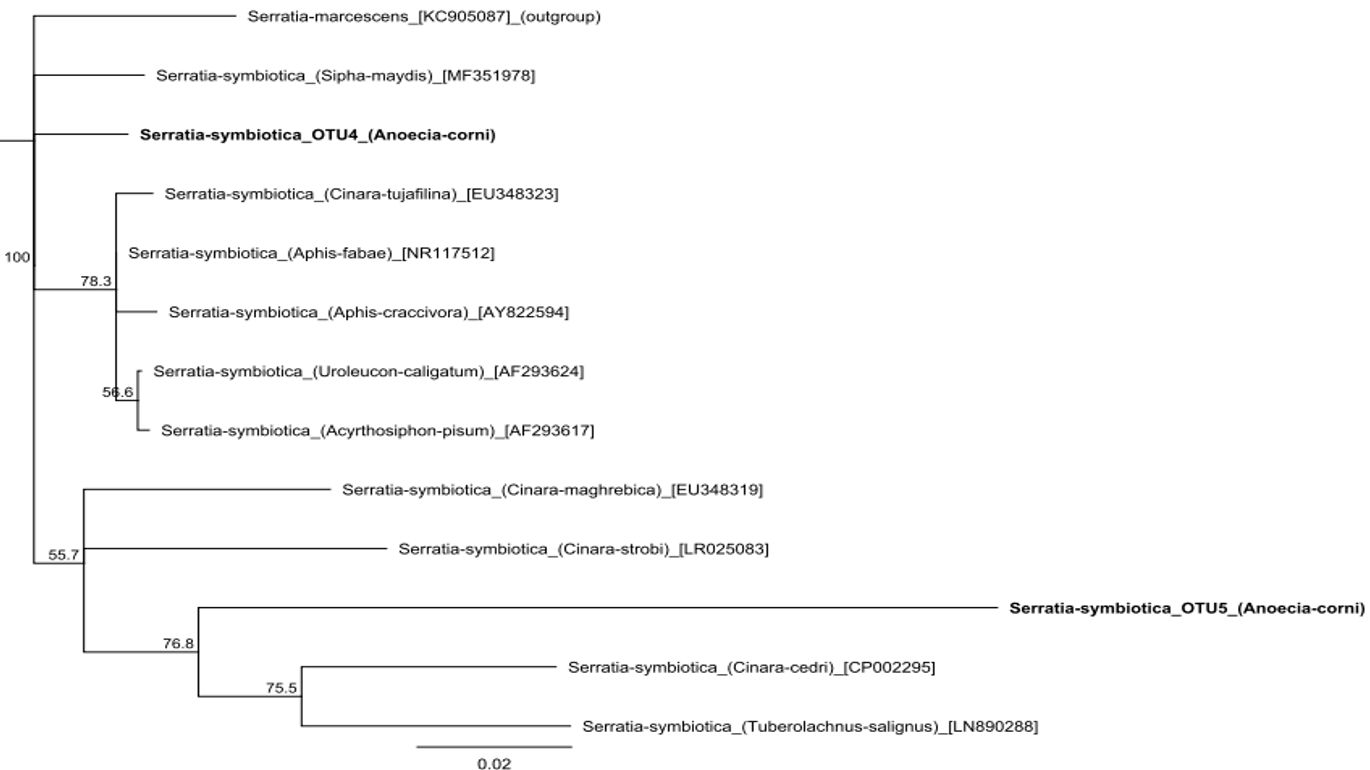

Supplement: S2 Fig — The evolutionary history was inferred using the Neighbor Joining (NJ) methods, with a J-C model. The percentage of replicate trees was verified with bootstrap of 1000 replicates. Designation in bold are is strains sequenced in this study. Host names are followed by the GenBank accession number of each bacterial sequence. Geneious version 6.1 created by Biomatters. (TIF) [file pone.0256019.s002.tif]

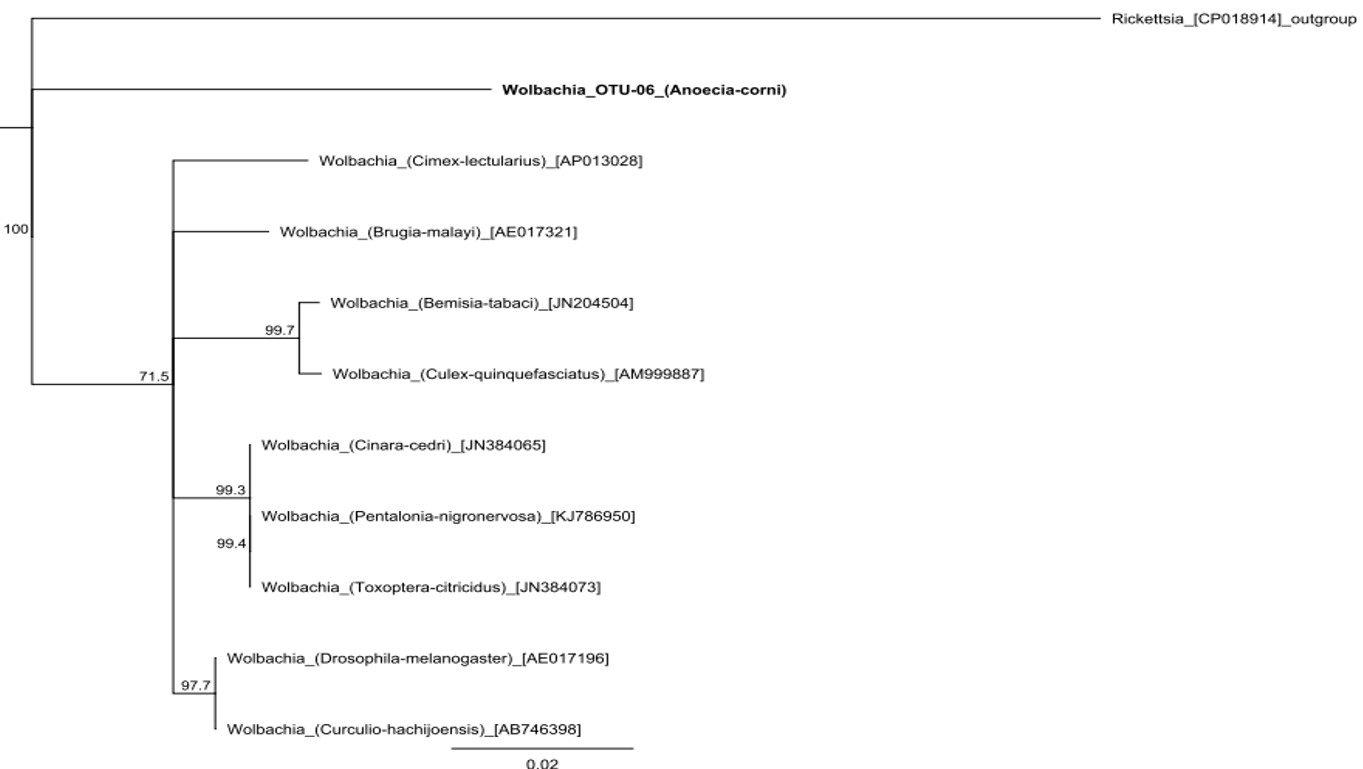

Supplement: S3 Fig — The evolutionary history was inferred using the Neighbor Joining (NJ) methods, with a HKY model. The percentage of replicate trees was verified with bootstrap of 1000 replicates. Designation in bold is strain sequenced in this study. Host names are followed by the GenBank accession number of each bacterial sequence. Geneious version 6.1 created by Biomatters. (TIF) [file pone.0256019.s003.tif]

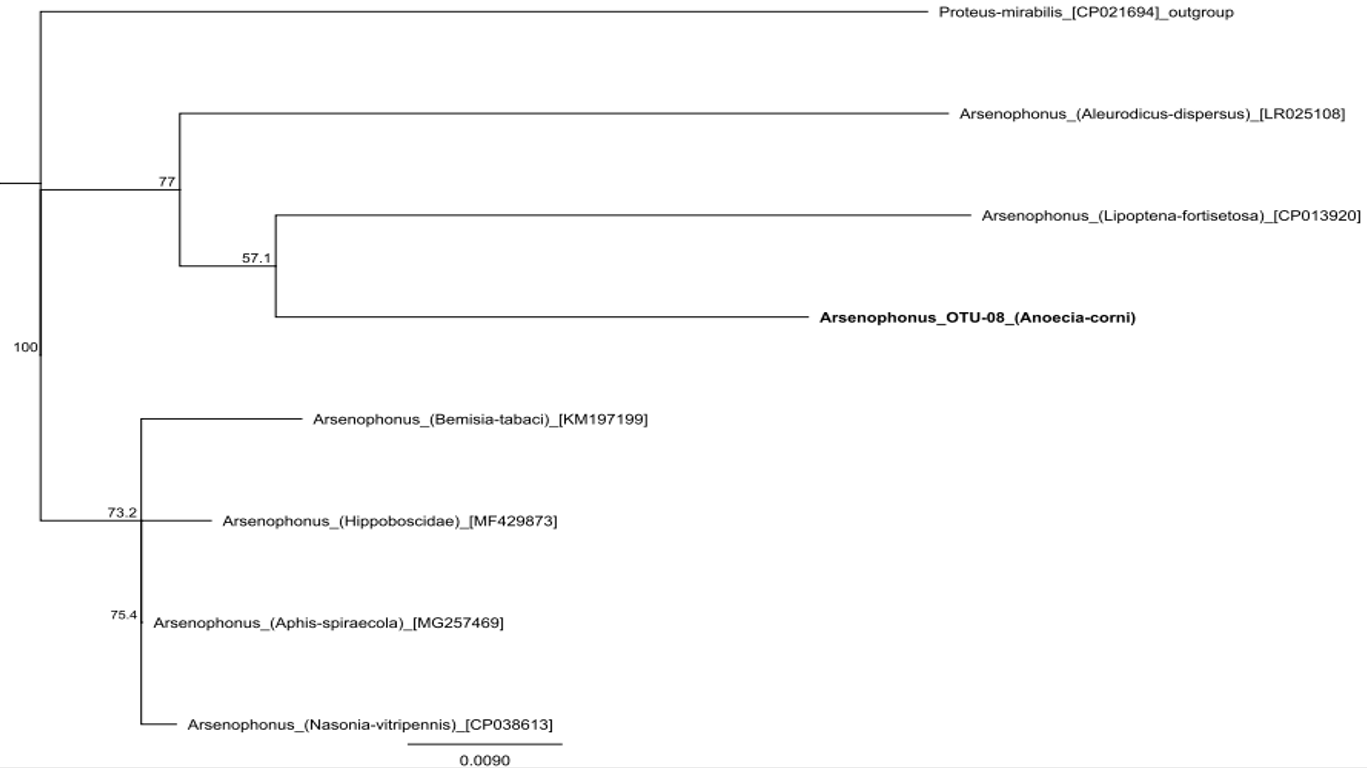

Supplement: S4 Fig — The evolutionary history was inferred using the Neighbor Joining (NJ) methods, with a HKY model. The percentage of replicate trees was verified with bootstrap of 1000 replicates. Designation in bold is strain sequenced in this study. Host names are followed by the GenBank accession number of each bacterial sequence. Geneious version 6.1 created by Biomatters. (TIF) [file pone.0256019.s004.tif]
