# Supplementary material for: Insight into the bacterial communities of the subterranean aphid Anoecia corni
Source: PLoS One. 2021 Aug 11;16(8):e0256019. doi: 10.1371/journal.pone.0256019 (PMC8357138; doi:10.1371/journal.pone.0256019)
Supplement: S1 Table — (DOCX) [file pone.0256019.s005.docx]

**Table S1.** **Summary of collection details and 16S rRNA gene sequencing results for aphid samples.**

| **Colony no.** | **Sample Accession ENA** | **Aphid species** | **Host plant** | **Region** | **Geographical coordinates** | **Date** | **Number of reads after quality filtering** |
| --- | --- | --- | --- | --- | --- | --- | --- |
| A1 | SAMEA6372654 | *A. corni* | Wheat | Casablanca-Settat | 33.045125 -7.572702 | 08/2014 | 99,318 |
| B1 | [SAMEA6372656](https://www.ebi.ac.uk/ena/browser/view/SAMEA6372656) | *A. corni* | Wheat | Casablanca-Settat | 33.304900 -7.190927 | 08/2014 | 171,158 |
| C1 | [SAMEA6372660](https://www.ebi.ac.uk/ena/browser/view/SAMEA6372660) | *A. corni* | Wheat | Casablanca-Settat | 33.541726 -7.458040 | 08/2014 | 180,823 |
| D1 | [SAMEA6372662](https://www.ebi.ac.uk/ena/browser/view/SAMEA6372662) | *A. corni* | Wheat | Casablanca-Settat | 32.605809 -8.370097 | 08/2014 | 110,089 |
| E1 | SAMEA6372664 | *A. corni* | Wheat | Casablanca-Settat | 33.322856 -7.140526 | 08/2014 | 192,532 |
| F1 | SAMEA6372666 | *A. corni* | Wheat | Casablanca-Settat | 32.815643 -7.699729 | 08/2014 | 198,089 |
| G1 | SAMEA6372667 | *A. corni* | Wheat | Casablanca-Settat | 32.471022 -7.194507 | 08/2014 | 169,665 |
| H1 | SAMEA6372669 | *A. corni* | Wheat | Casablanca-Settat | 32.858513 -8.225716 | 08/2014 | 166,061 |
| A2 | [SAMEA6372655](https://www.ebi.ac.uk/ena/browser/view/SAMEA6372655) | *A. corni* | Wheat | Béni Mellal-Khénifra | 32.525256 -6.948238 | 08/2014 | 126,972 |
| B2 | [SAMEA6372657](https://www.ebi.ac.uk/ena/browser/view/SAMEA6372657) | *A. corni* | Wheat | Béni Mellal-Khénifra | 32.904779 -6.827970 | 08/2014 | 127,484 |
| C2 | [SAMEA6372661](https://www.ebi.ac.uk/ena/browser/view/SAMEA6372661) | *A. corni* | Wheat | Béni Mellal-Khénifra | 33.186102 -6.211823 | 08/2014 | 141,700 |
| D2 | SAMEA6372663 | *A. corni* | Wheat | Béni Mellal-Khénifra | 32.466164 -5.842378 | 08/2014 | 82,338 |
| E2 | SAMEA6372665 | *A. corni* | Wheat | Béni Mellal-Khénifra | 31.752711 -6.993677 | 08/2014 | 132,175 |
| G2 | SAMEA6372668 | *A. corni* | Wheat | Béni Mellal-Khénifra | 32.145730 -6.376530 | 08/2014 | 141,049 |
| H12 | SAMEA6372670 | *A. corni* | Wheat | Béni Mellal-Khénifra | 33.148221 -5.568350 | 08/2014 | 196,477 |
| H2 | SAMEA6372671 | *A. corni* | Wheat | Béni Mellal-Khénifra | 32.309962 -6.339487 | 08/2014 | 173,752 |
| **Total** |  |  |  |  |  |  | 2,409,682 |
