# Supplementary material for: Insight into the bacterial communities of the subterranean aphid Anoecia corni
Source: PLoS One. 2021 Aug 11;16(8):e0256019. doi: 10.1371/journal.pone.0256019 (PMC8357138; doi:10.1371/journal.pone.0256019)
Supplement: S2 Table — (DOCX) [file pone.0256019.s006.docx]

Table S2. Data analysis:

Commands and options used to build the OUT table.

Load R packages

suppressPackageStartupMessages({

**library**(Biostrings)

**library**(ape)

**library**(dada2)

})

Merge paired-end reads using the fasq_mergepairs function

listfiles <- list.files('00-reads/',full.names = T)

forward <- listfiles[grep('_R1',listfiles)]

reverse <- listfiles[grep('_R2',listfiles)]

samplename <- substr(basename(forward),1,3)

nfiles <- length(forward)

**for**(i **in** 1:nfiles)

{

myarg <- paste0('-fastq_mergepairs ',forward[i],' -reverse ',reverse[i],' -fastqout 01-merged-pairs/',samplename[i],'.fastq',' -report 01-merged-pairs/report_',samplename[i],'.txt',' -fastq_maxdiffs 10 -fastq_pctid 80')

system2(command='./usearch11.0.667_i86linux32' ,args=myarg,stdout=F)

}

Quality filtering

summary.filter <- matrix(nrow=nfiles,ncol=2)

**for**(i **in** 1:nfiles)

{

myarg <- paste('-fastq_filter 01-merged-pairs/',samplename[i],'.fastq -fastq_minlen 450 -fastq_maxee 1 -fastaout 02-qual-filter/',samplename[i],'.fasta -relabel ',samplename[i],'.',sep='')

out <- system2(command='./usearch11.0.667_i86linux32' ,args=myarg,stdout=T,stderr=TRUE)

summary.filter[i,1] <- as.numeric(strsplit(gsub(" ", "", out[grepl('Reads',out)], fixed = TRUE),split='Reads')[[1]][1])

summary.filter[i,2] <- as.numeric(strsplit(gsub(" ", "",out[grepl('Filtered',out)], fixed = TRUE),split='Filtered')[[1]][1])

}

summary.filter <- data.frame(samplename,summary.filter)

colnames(summary.filter) <- c('samplename','before quality filter','after quality filter')

Pooling of the fasta sequences of all samples into a single fasta file

myarg <- ''

**for**(i **in** 1:nfiles)

{myarg <- paste(myarg,'02-qual-filter/',samplename[i],'.fasta ',sep='')}

myarg <- paste(myarg,'> 03-merged-sample/merged.fasta',sep='')

system2(command='cat',args=myarg)

Dereplication and sorting by size

myarg <- paste('-fastx_uniques 03-merged-sample/merged.fasta -fastaout 04-dereplicated/derep.fasta -sizeout -relabel Uniq')

system2(command='./usearch11.0.667_i86linux32',args=myarg)

myarg <- paste('-sortbysize 04-dereplicated/derep.fasta -fastaout 05-sorted/sorted.fasta -minsize 2')

system2(command='./usearch11.0.667_i86linux32',args=myarg)

OTU Clustering

myarg <- paste('-cluster_otus 05-sorted/sorted.fasta -otus 06-otus/otus.fasta -uparseout 06-otus/result.txt -relabel OTU_')

system2(command='./usearch11.0.667_i86linux32',args=myarg)

otus <- readDNAStringSet("06-otus/otus.fasta")

notus <- length(otus)

names(otus) <- sprintf("OTU_%03d", seq(1,notus))

writeXStringSet(otus,'06-otus/otus_renamed.fasta',format='fasta')

Mapping

myarg <- paste('-usearch_global 03-merged-sample/merged.fasta -db 06-otus/otus_renamed.fasta -strand plus -id 0.97 -uc 07-mapping/readmap.uc')

system2(command='./usearch11.0.667_i86linux32',args=myarg)

myarg <- paste('00_pythonscript/uc2otutab.py 07-mapping/readmap.uc')

system2(command='./00_CONDA_python/bin/python',args=myarg,stdout='07-mapping/tab.txt')

Discarding of lowly abundant OTU (<0,005 %)

tab1 <- read.table('07-mapping/tab.txt',header=T)

tab1 <- tab1[sort.list(tab1$OTUId),]

tabreduced <- tab1[apply(tab1[,-1],1,sum)/sum(apply(tab1[,-1],1,sum))>0.00005,]

otus <- readDNAStringSet("06-otus/otus_renamed.fasta", format = "fasta")

otus <- otus[is.element(names(otus),tabreduced$OTUId)]

length(otus)

## [1] 23

Taxonomy Assignment

otus.character <- as.character(otus)

taxa <- assignTaxonomy(otus.character, "00-greengenes-for-dada2/gg_13_8_train_set_97.fa", multithread=FALSE)
