# Supplementary material for: Insight into the bacterial communities of the subterranean aphid Anoecia corni
Source: PLoS One. 2021 Aug 11;16(8):e0256019. doi: 10.1371/journal.pone.0256019 (PMC8357138; doi:10.1371/journal.pone.0256019)
Supplement: S3 Table — (DOCX) [file pone.0256019.s007.docx]

**Table S3. Bacterial representative sequences (operational taxonomic units) found in *A. corni*.**

| **OTU Id** | **Read counts** | **PC reads** | **OTUs** |
| --- | --- | --- | --- |
| OTU 1 | 1,416,170 | 58.77 | >OTU1_*Buchnera aphidicola*  CCTACGGGTG GCAGCAGTGG GGAATATTGC ACAATGGGCG AAAGCCTGAT GCAGCTATGC  CGCGTGTATG AAGAAGGCCT TAGGGTTGTA AAGTACTTTC AGCAGGGAGG AAAGAAATAT  GTTTAATACA TATATTTTGT GACGTTACCT GCAGAAGAAG CACCGGCTAA CTCCGTGCCA  GCAGCCGCGG TAATACGGAG GGTGCAAGCG TTAATCAGAA TTACTGGGCG TAAAGAGCAC  GTAGGTGGTT TTTTAAGTCA GATGTGAAAT CCCTAGGCTT AACCTAGGAA CTGCATTTGA  AACTAATAGA CTAGAGTATC GTAGAGGGAG GTAGAATTCT AGGTGTAGCG GTGAAATGCG  TAGATATCTA GAGGAATACC TGTGGCGAAA GCGACCTCCT AAACGAATAC TGACACTGAG  GTGCGAAAGC GTGGGGAGCA AACAGGATTA GATACCCCTG TAGTC |
| OTU 2 | 483,864 | 20.08 | >OTU2_*Buchnera aphidicola*  CCTACGGGTG GCAGCAGTGG GGAATATTGC ACAATGGGCG AAAGCCTGAT GCAGCTATGC  CGCGTGTATG AAGAAGGCCT TAGGGTTGTA AAGTACTTTC AGCGGGGAGG AAAGAGAAAA  TATTTAATAA ATATTATCTT ATGACGTTAC CCGAATAAGA AGCACCGGCT AACTCCGTGC  CAGCAGCCGC GGTAATACGG AGGGTGCTAG CGTTAATCAG AATTACTGGG CGTAAAGAGC  ACGTAGGCGG TTTTTTAAGT CAGATGTGAA ATCCCTGGGC TTAACCTAGG AACTGCATTT  GAAACTGGAA GGCTAGAGTA TCGTAGAGGG AGGTAGAATT CTAGGTGTAG CGGTGAAATG  CGTAGATATC TAGAGGAATA CCTGTGGCGA AAGCGGCCTC CTAAACGAAT ACTGACGCTG  AGGTGCGAAA GCGTGGGGAG CAAACAGGAT TAGATACCCC TGTAGTC |
| OTU 3 | 417,839 | 17.34 | OTU3_*Buchnera aphidicola*  CCTACGGGTG GCAGCAGTGG GGAATCTTGC ACAATGAGCG AAAGCTTGAT GCAGCTATGC  CGCGTGTATG AAGAAGGCCT TAGGGTTGTA AAGTACTTTC AGTAGAGAAG AAGAAATAAA  AAATAATACA TTTTATTTTT GACGTTATTT ACAAAAGAAG CACCGGCTAA CTCCGTGCCA  GCAGCCGCGG TAATACGGAG GGTGCTAGCG TTAATCAGAA TTACTGGGCG TAAAGAGCAC  GTAGGTGGTT TTTTAAGTCA GATGTGAAAT CCCTAGGCTT AACCTAGGAA CTGCATTTGA  AACTAAAAAA CTAGAGTTTC ATAGAGGGAG GTAGAATTCT AGGTGTAGCG GTGAAATGCG  TAGATATCTG GAGGAATACC TGTGGCGAAA GCGGCCTCCT AAATGATAAC TGACACTGAG  GTGCGAAAGC ATGGGGAGCA AACAGGATTA GATACCCCAG TAGTC |
| OTU 4 | 63,134 | 2.62 | OTU4_ *Serratia symbiotica*  CCTACGGGTG GCAGCAGTGG GGAATATTGC ACAATGGGCG CAAGCCTGAT GCAGCCATGC  CGCGTGTGTG AAGAAGGCCT TCGGGTTATA AAGCACTTTC AGCGAGGAGG AAGGGTAATG  TGTTAATAAG ACATTGCATT GACGTTACTC GCAGAAGAAG CACCGGCTAA CTCCGTGCCA  GCAGCCGCGG TAATACGGAG GGTGCAAGCG TTAATCGGAA TTACTGGGCG TAAAGCGCAC  GCAGGCGGTT TGTTAAGTCA GATGTGAAAT CCCCGCGCTC AACGTGGGAA CGGCATTTGA  GACTGGCAAG CTAGAGTCTT GTAGAGGGGG GTAGAATTCC AGGTGTAGCG GTGAAATGCG  TAGAGATCTG GAGGAATACC GGTGGCGAAG GCGGCCCCCT GGACAAAGAC TGACGCTCAGGTGCGAAAGC GTGGGGAGCA AACAGGATTA GATACCCCTG TAGTC |
| OTU 5 | 21,446 | 0.89 | OTU5_ *Serratia symbiotica*  CCTACGGGGG GCAGCAGTGG GGGATATTGC ACAATGGGGG AAACCCTGAT GCAGCCATGC  CGCGTGTATG AAGAAGGCTT TCGGGTTGTA AAGTACTTTC GGTAATGAGG AAGGTGTATT  ATCTAACAGG TAATGCAATT GACGTTAGTT ACAGAAGAAG CACCGGCTAA CTCCGTGCCA  GCAGCCGCGG TAATACGGAG GGTGCGAGCG TTAATCGGAA TAACTGGGTG TAAAGGGCAT  GCAGGCGGGT CATTAAGTTA GGTGTGAAAT CCCCGGGCTC AACCTGGGAA CTGCACTTAA  AACTGGTGGT CTGGAGTATT GTAGAGGAAG GTAGAATTCC ACGTGTAGCG GTGAAATGCG  TAGAGATGTG GAGGAATACC GGTGGCGAAG GCGGCCTTCT GGACAAATAC TGACGCTGAG  ATGCGAAAGC GTGGGGAGCA AACAGGATTA GATACCCCTG TAGTC |
| OTU 6 | 964 | 0.04 | OTU6_ *Wolbachia*  CCTACGGGGG GCAGCAGTGG GGAATATTGC ACAATGGGCG AAAGCCTGAT GCAGCTATGC  CGCGTGTATG AAGAAGGCCT TAGGGTTGTA AAGTACTTTC AGCGGGGAGG AAAGAGAAAA  TATTTAATAA ATATTATCTT ATGACGTTAC CCGAATAAGA AGCACCGGCT AACTCCGTGC  CAGCAGCCGC GGTAATACGG AGAGGGCTAG CGTTATTCGG AATTATTGGG CGTAAAGAGC  GCGTAGGCTG ATTAGTAAGT TAAAAGTGAA ATCCCAAAGC TTAACTTTGG AATTGCTTTT  AAAACTGCTA ATCTAGAGAT TGAAAGAGGA TAGAGGAATT CCTAGTGTAG AGGTGAAATT  CGTAAATATT AGGAGGAACA CCAGTGGCGA AGGCGTCTAT CTGGTTCAAA TCTGACGCTG  AGGCGCGAAG GCGTGGGGAG CAAACAGGAT TAGATACCCC TGTAGTC |
| OTU 7 | 241 | 0.01 | OTU7_*Buchnera aphidicola*  CCTCCTTTTT TCCTCCTTTT TTCCTCTTTC CCCCTTTTCT CCCTCCTTCT TCCTCTCTTC  CTCTTTTCTT CCTAAGGCCT TAGGGTTGTA AAGTACTTTC AGCAGGGAGG AAAGAAATAT  GTTTAATACA TATATTTTGT GACGTTACCT GCAGAAGAAG CACCGGCTAA CTCCGTGCCA  GCAGCCGCGG TAATACGGAG GGTGCAAGCG TTAATCAGAA TTACTGGGCG TAAAGAGCAC  GTAGGTGGTT TTTTAAGTCA GATGTGAAAT CCCTAGGCTT AACCTAGGAA CTGCATTTGA  AACTAATAGA CTAGAGTATC GTAGAGGGAG GTAGAATTCT AGGTGTAGCG GTGAAATGCG  TAGATATCTA GAGGAATACC TGTGGCGAAA GCGACCTCCT AAACGAATAC TGACACTGAG  GTGCGAAAGC GTGGGGAGCA AACAGGATTA GATACCCCTG TAGTC |
| OTU 8 | 241 | 0.01 | OTU8_*Arsenophonus*  CCTACGGGGG GCAGCAGTGG GGAATATTGC ACAATGGGCG AAAGCCTGAT GCAGCCATGC  CGCGTGTATG AAGAAGGCTT TCGAGTTGTA AAGTACTTTC AGTCGTGATG AAGGTGTTAA  GATTAATAAT CTTAACAATT GACATTAGCG AAAGAAGAAG CACCGGCTAA CTCCGTGCCA  GCAGCCGCGG TAATACGGAG GGTGCGAGCG TTAATCGGAA TTACTGGGCG TAAAGGGCAC  GCAGGCGGTT AATTAAGTTG GATGTGAAAG CCCTAGGCTC AACCTAGGAA TGGCATCCAA  TACTGGTTAG CTAGAGTTTT GTAGAGGGGG GTAAAATTCC ATGTGTAGCG GTGAAATGCG  TAGAGATATG GAGGAATACC AGTGGCGAAG GCGGCCCCCT GGACAAAAAC TGACGCTCAT  GTGCGAAAGC GTGGGGAGCA AACAGGATTA GATACCCTAG TAGTC |
| OTU 9 | 241 | 0.01 | OTU9_ *Dactylopiibacterium carminicum*  CCTACGGGGG GCAGCAGTGA GGAATTTTGG ACAATGGGGG AAACCCTGAT CCAGCCATGC  CGCGTGAGTG AAGAAGGCCT TCGGGTTGTA AAGCTCTTTC AGTCGGAAAG AAATGGTTTC  GGTGAATATC CGAAGCCGAT GACGGTACCG ACAAAAGAAG CACCGGCTAA CTACGTGCCA  GCAGCCGCGG TAATACGTAG GGTGCGAGCG TTAATCGAAA TTACTGGGCG TAAAGCGTGC  GCAGGCGGTT GTGCAAGACA GGTGTGAAAT CCCCAGGCTT AACCTGGGAA CTGCGCTTGT  GACTGCACGA CTAGAGTACG GCAGAGGGGG GTGGAATTCC TGGTGTAGCA GTGAAATGCG  TAGATATCAG GAGGAACATC GATGGCGAAG GCAGCCCCCT GGGCCTGTAC TGACGCTCAT  GCACGAAAGC GTGGGGAGCA AACAGGATTA GATACCCCTG TAGTC |
| OTU 10 | 241 | 0.01 | OTU10_ *Buchnera aphidicola*  GACTACTCGG GTATCTAATC CTGTTTGCTC CCCACACTTT CGCACCTCAG TGTCAGTATT  CGTTTAGGAG GTCGCTTTCG CCACAGGTAT TCCTCTAGAT ATCTACGCAT TTCACCGCTA  CACCTAGAAT TCTACCTCCC TCTACGATAC TCTAGTCTAT TAGTTTCAAA TGCAGTTCCT  AGGTTAAGCC TAGGGATTTC ACATCTGACT TAAAAAACCA CCTACGTGCT CTTTACGCCC  AGTAATTCTG ATTAACGCTT GCACCCTCCG TATTACCGCG GCTGCTGGCA CGGAGTTAGC  CGGTGCTTCT TCTGCAGGTA ACGTCACAAA ATATATGTAT TAAACATATT TCTTTCCTCC  CTGCTGAAAG TACTTTACAA CCCTAAGGCC TTCTTCATAC ACGCGGCATA GCTGCATCAG  GCTTTCGCCC ATTGTGCAAT ATTCCCCACT GCTGCCCCCC GTAGG |
